# The Effect of Natural Chitosan, Humate and Their Complexes on the Rheological Properties of Wet Kaolinite †

**DOI:** 10.3390/polym18212690

**Published:** 2026-10-30

**Authors:** Aleksandr A. Kiushov, Irina G. Panova, Vyacheslav S. Molchanov, Maxim S. Arzhakov, Leonid O. Iliasov, Olga E. Philippova, Alexander A. Yaroslavov

**Affiliations:** 1 Department of Chemistry, M.V. Lomonosov Moscow State University, Leninskie Gory 1, 119991 Moscow, Russia; 2 Department of Physics, M.V. Lomonosov Moscow State University, Leninskie Gory 1, 119991 Moscow, Russia; 3 Key Laboratory of Advanced Textile Materials and Manufacturing Technology, Zhejiang Sci-Tech University, Hangzhou 310018, China

**Keywords:** clay, kaolinite, potassium humates, chitosan, polycomplex, rheology

## Abstract

Control of mechanical properties of natural clays and clayey soils under saturation with water is an important task in agriculture and geoengineering. In this study, kaolinite was modified with cationic chitosan, anionic potassium humate (PHum), and non-stoichiometric chitosan–PHum interpolyelectrolyte complexes (IPCs). The optimal composition of IPCs in terms of the ratio between positively charged chitosan groups and negatively charged potassium humate groups, Q = [${\mathrm{NH}}_{3}^{+}$]/[COO^−^], was determined using laser microelectrophoresis, turbidimetry, and dynamic light scattering and showed Q ≤ 0.3 for anionic IPCs and Q ≥ 3 for cationic IPCs. Amplitude tests showed that introduction of minor amounts of chitosan, not more than 0.5 wt.%, to kaolinite resulted in increased strength characteristics of wet clay by 0.5–1.5 orders of magnitude, which was due to the formation of the cooperative physical network via electrostatic interaction between positively charged chitosan and negatively charged clay particles. Binding of the same amounts of anionic PHum to kaolinite decreased the strength parameters by 0.5–1.0 orders of magnitude because of increased sliding of the clay particles. Addition of 0.25 wt.% of both cationic and anionic IPCs to kaolinite allowed the preservation of the reinforcing effect of chitosan and suppression of the weakening action of PHum. Such behavior of chitosan–PHum IPCs was likely due to redistribution of cationic chitosan between anionic PHum and kaolinite and formation of the kaolinite–chitosan–PHum ternary complex, which minimizes the destabilizing effect of PHum and improves the mechanical properties of the clay.

## 1. Introduction

Aluminosilicates—montmorillonite, bentonite, etc.—are the inherent and essential components of soil and the ground and contribute significantly to their structural stability and resistance to erosion [1,2]. These minerals are widely used in construction, mining, urban and oil/gas industries [3,4,5] as well as for the production of modern composite materials [6,7,8,9]. One of the main drawbacks of these materials is their pronounced water absorption capacity, which leads to a sharp decrease in their rheological and mechanical characteristics such as viscosity, shear stress and storage modulus [10,11,12]. In connection with this, increasing the water resistance of natural clays and clayey soils is considered as a challenging problem associated with expanding their practical application.

To solve this problem, polymeric additives are widely used to control the rheological behavior of diluted and concentrated clay suspensions [13,14,15,16,17]. The structural and mechanical properties of modified dispersed soils are controlled by the polymer nature, chemical structure, molar-mass characteristics and application rate as well as the type of soil and the moisture content [17,18,19,20,21]. For example, high molecular mass ionic polymers (polyelectrolytes, PEs) promote particle flocculation via the bridging mechanism, thereby improving rheological properties of dispersions.

Recently, interpolyelectrolyte complexes (IPCs)—products of the interaction of cationic and anionic polymers—have been proposed as soil modifiers [22,23,24,25]. The most promising recipes involve water-soluble non-stoichiometric IPCs [25,26,27]. The amphiphilic nature and controlled composition of IPCs make them effective soil binders and anti-erosion agents [25] as well as the inherent component of artificial soils (“constructozems”) [28]. Note that most of the formulations used are based on the combination of synthetic well-characterized polycations and polyanions with a clearly defined chemical composition.

At the present time, native PEs (anionic potassium humate (PHum) being the prime example) are of particular interest due to their biostimulating and immunomodulatory properties [29,30]. Humates are the inherent additives to artificial soils and low-fertility soils. However, humates reduce the viscosity of clay suspensions and promote dispersion of wet soils, and, as a result, mechanical characteristics of the material worsen [31,32,33].

Earlier [28,34,35,36], complexation of humates with synthetic polycation (polydiallyldimethylammonium chloride (PDADMAC)) was shown to neutralize the weakening effect of humates [34] when their biological activity was retained. In other words, IPCs based on synthetic polycation (PDADMAC) and natural polyanion (PHum) provided simultaneous increase in mechanical properties and soil fertility.

Note that a current challenge in polymer material science is associated with the replacement of synthetic products with natural analogs. Taking into account this trend, in this paper, physicochemical regularities of the complexation of natural PEs—anionic PHum and cationic chitosan—were studied. The latter was chosen due to its commercial availability, biocompatibility and biodegradability. In agriculture, chitosan is used for bioremediation of contaminated soils and as an immunostimulant for plants [37,38,39].

The aim of the work involved the study of the regimes of the (1) preparation of non-stoichiometric water-soluble chitosan/PHum IPCs, (2) modification of wetted kaolinite with PHum, chitosan and their IPCs, and (3) effect of these natural modifiers on the rheological behavior of kaolinite pastes.

## 2. Materials and Methods

### 2.1. Materials

Kaolinite (Sigma-Aldrich, Shanghai, China), PHum with M_w_ = 9.9 kDa (Humintech GmbH, Grevenbroich, Germany), and chitosan with M_η_ = 50–190 kDa (Sigma-Aldrich, Shanghai, China) were used as received. Acetic acid (70% aqueous solution), hydrochloric acid (37% aqueous solution), disodium hydrogen phosphate, sodium dihydrogen phosphate, potassium chloride (all chemicals from Chimmed, Moscow, Russia), and sodium hydroxide (Riedel-de Haën, Seelze, Germany) were of analytical grade and used without additional purification.

The content of NH_2_ groups and ${\mathrm{NH}}_{3}^{+}$ groups at pH = 5.3 in chitosan was determined by potentiometric titration in accordance with [40] using an Aquilon pH-420 pH-meter (Aquilon, Moscow, Russia) with a combination glass electrode HI 1131B at 22 °C and found to be 4.8 × 10^3^ mol·g^−1^. The content of COO^−^ groups in 0.01% PHum solution at pH = 5.3 was determined by potentiometric titration in accordance with [41] and found to be 0.224 mmol·L^−1^.

Chitosan and PHum were dissolved in acetate-phosphate buffer with pH = 5.3 ± 0.1 and ionic strength I = 0.19 mol·L^−1^. Initial buffer solution was obtained by mixing the solution of acetic acid with the solution of sodium dihydrogen phosphate. Resulting concentrations of the buffer components were 0.12 mol·L^−1^ and 0.07 mol·L^−1^ for acetic acid and sodium dihydrogen phosphate, respectively.

In order to obtain chitosan/PHum IPCs, the buffer solutions of both polymers were mixed in appropriate proportions. The composition of IPCs was characterized by the molar ratio of cationic chitosan groups and anionic PHum groups Q = [${\mathrm{NH}}_{3}^{+}$]/[COO^−^].

### 2.2. Methods

The hydrodynamic diameter of IPC particles in solutions was measured by dynamic light scattering using ZetaPlus (Brookhaven, Holtsville, NY, USA) at the scattering angle 90°; their electrophoretic mobility (EPM) was measured by laser microelectrophoresis in a thermostatic cell using ZetaPlus (Brookhaven, USA). The treatment of experimental results was carried out with the software provided by manufacturing companies.

Spectrophotometric analysis was performed using the spectrometer Genesys 50 (Thermo FS, Waltham, MA, USA) in a quartz cell at 22 °C and at a wavelength of 500 nm.

The rheology of both virgin and modified kaolinite samples was studied by a dynamic amplitude sweep in the oscillatory deformation mode [34,42,43] using a Physica MCR-301 rheometer (Anton Paar, Graz, Austria) with a measuring cell consisting of two parallel plates. The diameter of the upper rotating plate was of 2.5 cm, and the distance between the plates was of 3 mm. Testing was carried out at 20.0 ± 0.1 °C at a frequency of 0.5 Hz. For virgin kaolinite samples and kaolinite samples modified with chitosan and chitosan/PHum IPCs, the strain range was 0.001–100%. Kaolinite samples modified with PHum demonstrated more pronounced plasticity. In connection with this, their rheological behavior was studied within a broader strain range—0.001–300%.

The samples for rheological testing were prepared via addition of 5.8 mL of acetate-phosphate buffer or a solution of polymer modifiers in buffer to 8 g of air-dried kaolinite with a hygroscopic humidity of 3.5 ± 0.4%. After that samples were mechanically mixed for about 10 min and then kept at room temperature for 24 h until equilibrium was achieved [34]. The water content in pastes measured using Shimadzu MOC-63U (Shimadzu, Kyoto, Japan) was 78 ± 5%. The polymer content in the system was presented as wt.% relative to the weight of absolutely dry kaolinite.

All samples were prepared in triplicate. Statistical data processing was carried out in Excel. The results were expressed as mean ± confidence interval.

## 3. Results and Discussion

### 3.1. Formation of Chitosan/PHum Complexes

At pH 5.3 in aqueous solution, PHum macromolecules were negatively charged because of the dissociation of their carboxylic groups, while macromolecules of chitosan carried positive charges due to protonation of their amino groups. Mixing of both solutions led to interpolymer interaction and the formation of chitosan/PHum IPCs, stabilized by multiple salt bridges between the oppositely charged groups of both polymers [44,45].

**Figure 1:**
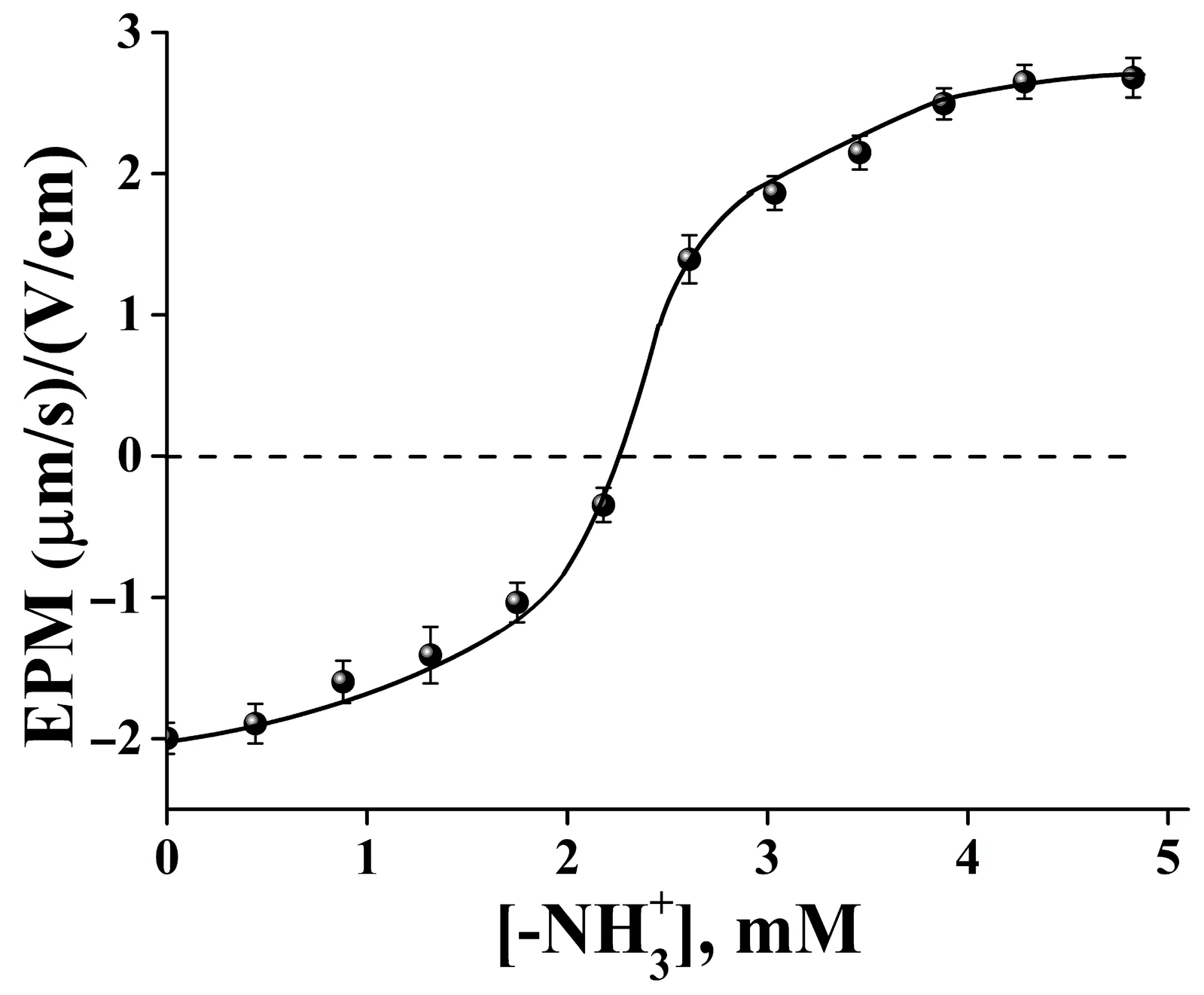
Electrophoretic mobility (EPM) of interpolyelectrolyte complexes in PHum/chitosan mixtures vs. chitosan concentration as [${\mathrm{NH}}_{3}^{+}$], 0.01 wt.% PHum solution, acetate-phosphate buffer, pH = 5.3 ± 0.1 (20 °C). Hereinafter error bars indicate the confidence intervals at *p* < 0.05.

The interaction was monitored via measuring the EPM of IPC particles in the system. Figure 1 shows how the EPM of IPC particles changes upon addition of increasing amounts of chitosan solution to 0.01 wt.% Phum solution with [COO^−^] = 0.224 mmol·L^−1^, while the chitosan concentration is expressed in moles of protonated amino groups [${\mathrm{NH}}_{3}^{+}$] per liter. Initially negatively charged PHum chains lose their charge when binding to chitosan; at [${\mathrm{NH}}_{3}^{+}$] = 0.22 mmol·L^−1^, an electroneutral IPC is formed with an equal amount of positive and negative charges. At higher chitosan concentrations, positively charged IPCs are obtained, whose EPM reaches a constant value at [${\mathrm{NH}}_{3}^{+}$] > 4 mmol·L^−1^. Hence, the ratios Q = [${\mathrm{NH}}_{3}^{+}$]/[COO^−^] < 1 and Q = [${\mathrm{NH}}_{3}^{+}$]/[COO^−^] > 1 are the criteria for the formation of non-stoichiometric anionic and cationic IPCs, respectively.

An important requirement for polycomplexes is their colloidal stability: such polycomplexes must be uniformly distributed in the kaolinite sample, which determines the reliability and reproducibility of rheological tests. The stability of IPCs against aggregation was examined using a traditional approach based on monitoring the turbidity of polymer solutions [26,46].

**Figure 2:**
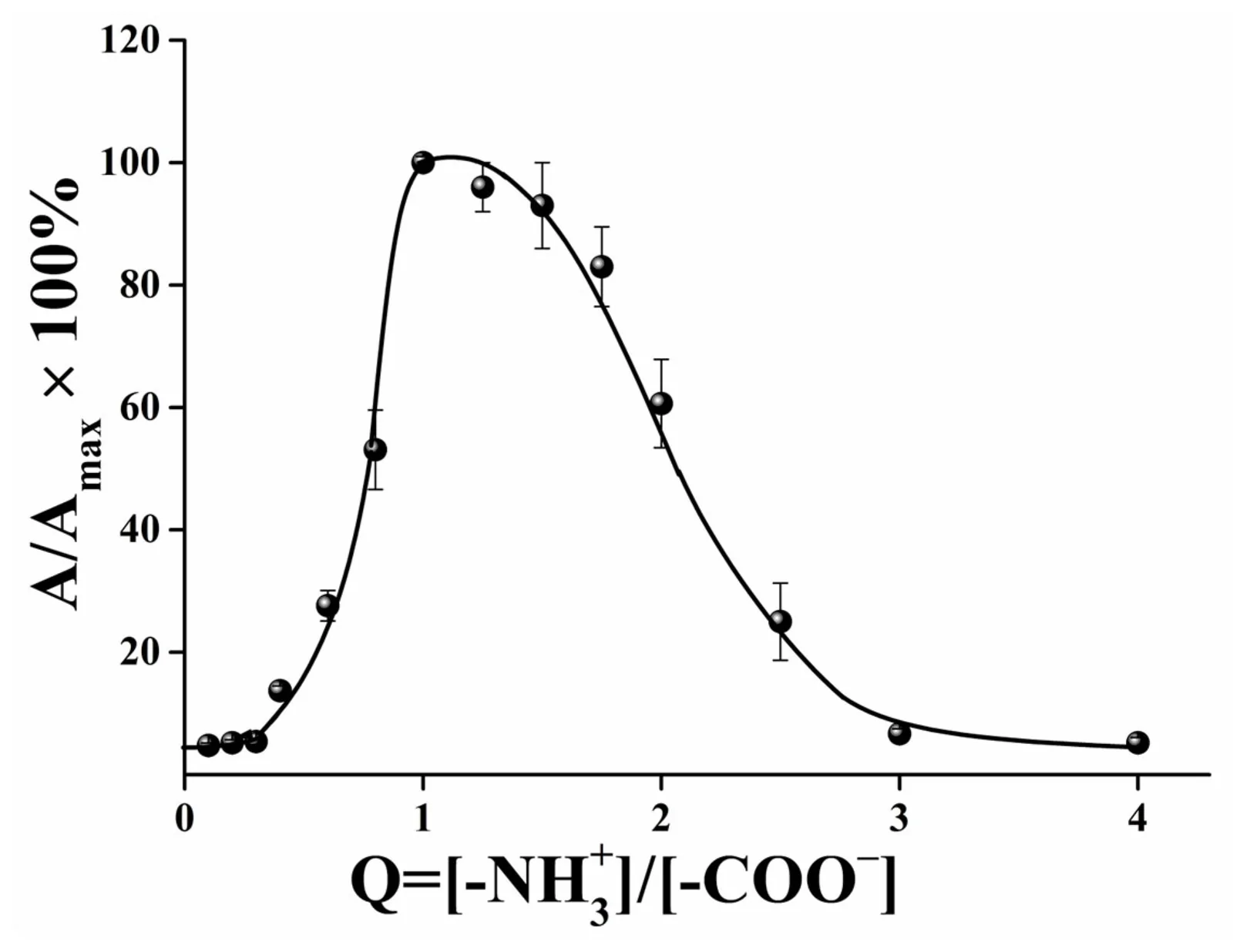
The dependences of relative optical density (A/A_max_ × 100%) of PHum/chitosan mixtures vs. the composition Q = [${\mathrm{NH}}_{3}^{+}$]/[COO^−^] at 500 nm. A total concentration of PHum and chitosan in mixtures was 0.01 wt.%, acetate-phosphate buffer, pH = 5.3 ± 0.1 (20 °C). Hereinafter error bars indicate the confidence intervals at *p* < 0.05.

Figure 2 shows the dependence of relative optical density of the IPC solution on Q = [${\mathrm{NH}}_{3}^{+}$]/[COO^−^]. Colloidally stable IPC solutions corresponded to a turbidity region not exceeding 10% of the maximum turbidity value [26,46]. This requirement was met for anionic IPC solutions with Q ≤ 0.3 and cationic IPC solutions with Q ≥ 3. Deviation from these Q values was accompanied by a sharp growth in optical density which reflected the aggregation of IPC particles. The maximum turbidity was observed at Q = 1, i.e., at a complete mutual neutralization of chitosan and PHum charges.

Taking into account the data presented in Figure 1 and Figure 2, the colloidal stability of non-stoichiometric IPCs is associated with abundant negative charges of PHum or positive charges of chitosan, which are not involved in the electrostatic interpolymer interactions.

Dynamic light scattering tests showed that the size (hydrodynamic diameter) of particles did not exceed 150 ± 30 nm in Q ≤ 0.3 IPC solutions and 800 ± 50 nm in Q ≥ 3 IPC solutions. These sizes have not changed for at least two weeks.

For modification of kaolinite, four chitosan/PHum IPCs were used. Their compositions and EPM values are given in Table 1. These IPCs contained hydrophobic sites composed by mutually neutralized chitosan and PHum units and free hydrophilic anionic or cationic segments. These IPCs are resistant to phase separation due to the high charge generated by the free polymer segments taken in excess.

### 3.2. Viscoelastic Properties of Original and Modified Kaolinite

**Figure 3:**
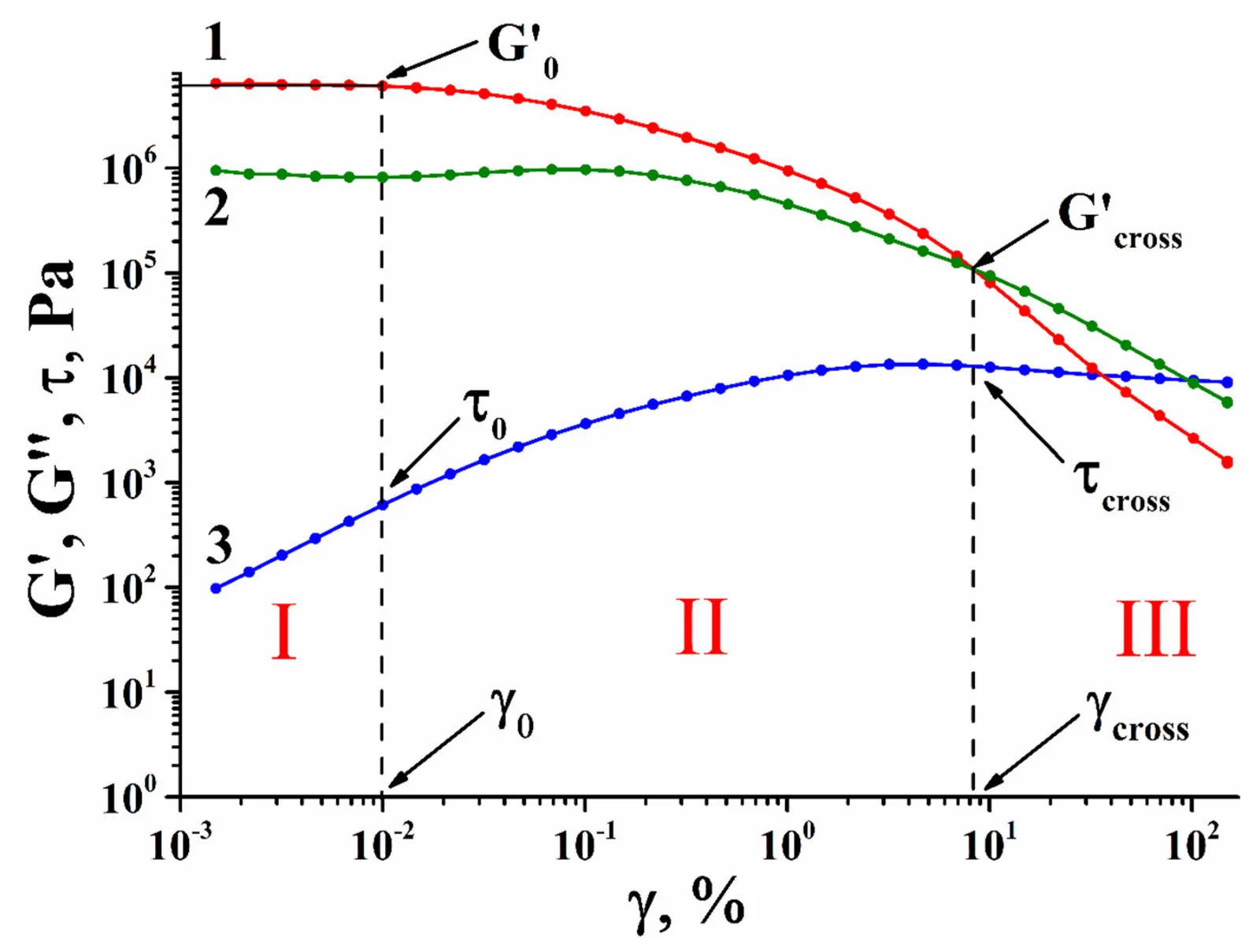
The strain (γ) dependences of storage modulus G′ (1), loss modulus G″ (2) and shear stress τ (3) for kaolinite modified with 0.25 wt.% of chitosan in logarithmic scale. I—region of linear viscoelasticity (γ < γ_0_); II—transition from linear viscoelasticity to flow (γ_0_ < γ < γ_cross_); III—region of steady-state flow (γ > γ_cross_). G′_0_ = τ_0_/γ_0_—initial storage modulus. G′_cross_ = τ_cross_⁄γ_cross_—storage modulus at cross-over point (G′ = G″).

Figure 3 shows the dependences of storage modulus G′ (curve 1), loss modulus G″ (curve 2), and shear stress τ (curve 3) on the strain γ for a kaolinite sample modified with 0.25 wt.% chitosan. The presented profiles are typical for all samples studied.

Within strain interval γ < γ_0_ (region I), a linear viscoelastic response of the sample is observed. The constant values of initial storage modulus G′_0_ and loss modulus G″_0_ evidence the stability of the clay structure, i.e., the preservation of interparticle bond networks under experimental conditions.

At strains γ > γ_0_ (region II), the decrease in both G′ and G″ is associated with the mechanically activated disruption of the above-mentioned physical network. This structural transformation is initiated by the shear stress τ_0_, and the pair τ_0_ and γ_0_ is identified as “yield point” [19,34,47].

With the further increase in strain, the dependences of G′ and G″ intersect at strain γ_cross_. At γ < γ_cross_, the elastic response of clay samples prevails (G′ > G″). At γ > γ_cross_ (region III), a steady-state flow of the samples is observed (G″ > G′) because of the complete destruction of the physical network initiated by the shear stress τ_cross_. The pair of parameters, τ_cross_ and γ_cross_, that are calculated in correspondence with the intersection of the G′ and G″ dependences can be defined as a “flow point”.

The strain interval γ_0_ < γ < γ_cross_ (region II, Figure 3) corresponds to the mechanically activated transition from linear viscoelasticity to steady-state flow [34].

**Figure 4:**
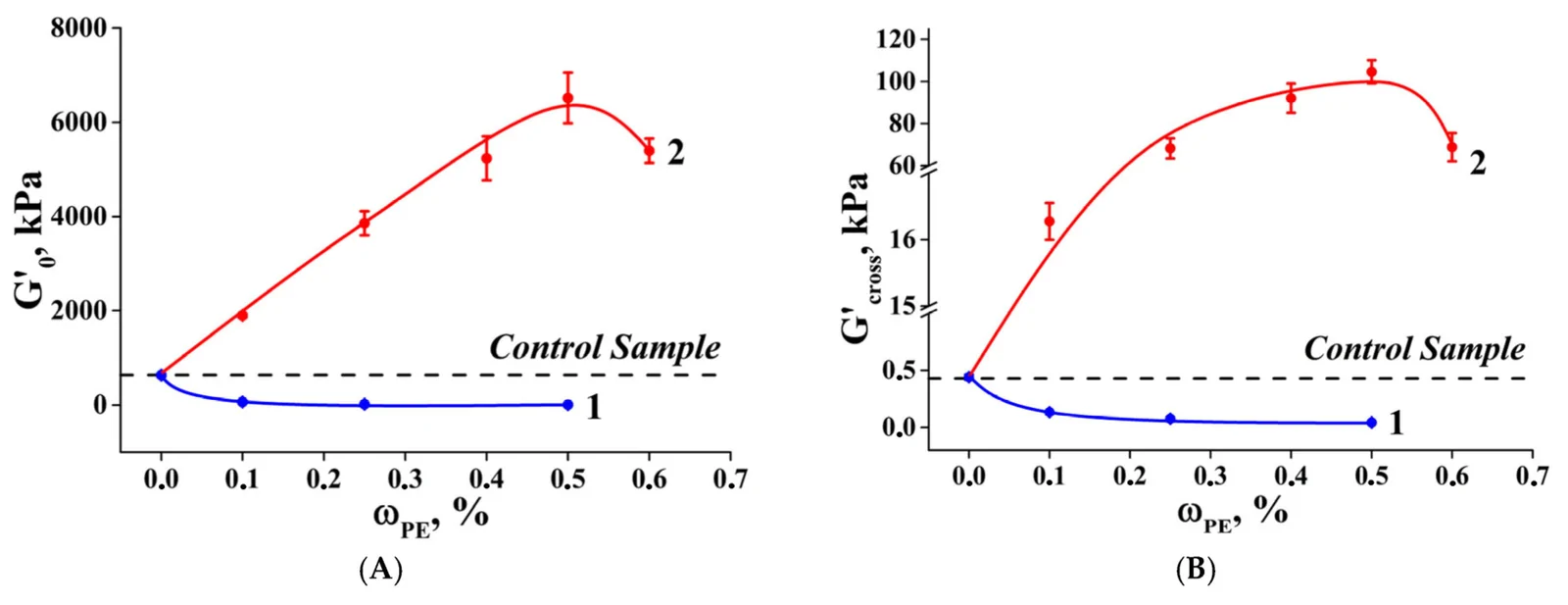

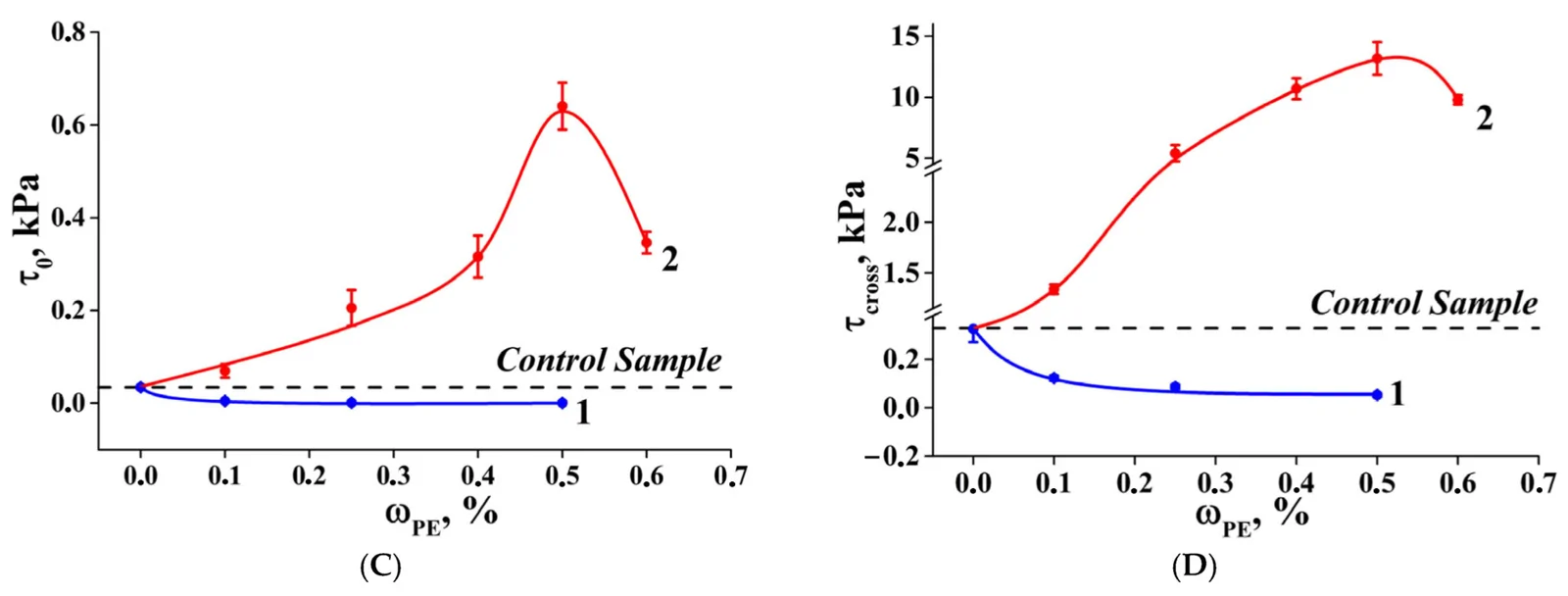
The dependences of G′_0_ (**A**), G′_cross_ (**B**), τ_0_ (**C**) and τ_cross_ (**D**) on polymer content for the samples: kaolinite–PHum (curves 1) and kaolinite–chitosan (curves 2). Horizontal dashed lines indicate the corresponding value of reference kaolinite–acetate-phosphate buffer-saturated sample (control sample). Hereinafter error bars indicate the confidence intervals at *p* < 0.05.

Hereinafter, to discuss the load response of both the original and modified kaolinite samples, the following strength characteristics are used: G′_0_, τ_0_, G′_cross_ and τ_cross_. Figure 4 shows the dependences of the above-mentioned four parameters on the weight polymer content (ω) for kaolinite–PHum (curves 1) and kaolinite–chitosan (curves 2) samples.

Modification of kaolinite with 0.1 wt.% PHum leads to the noticeable decrease in all of the above-mentioned rheological parameters (curves 1, Figure 4). At the following growth in the polymer content to 0.5 wt.%, these characteristics tend to constant values. It has been described previously that anionic humates interact with negatively charged clay platelets via ligand exchange, hydrogen bonding, metal cation bridging, and van der Waals and hydrophobic interactions [48,49]. PHum adsorption on the surface of kaolinite results in increasing its negative charge, stimulating electrostatic repulsion between kaolinite platelets and reducing the clay’s rheological parameters. An additional factor is the steric stabilization of clay particles by adsorbed humates [50,51].

For kaolinite modified with cationic chitosan, the dependences of the rheological characteristics on the polymer content go through the maximum at ω = 0.5 wt.% (curves 2, Figure 4). Positively charged chitosan chains interact with negatively charged clay particles, forming a cooperative physical network that improve the clay’s mechanical properties.

At a chitosan content of more than 0.5 wt.%, a decrease in rheological characteristics is observed, likely due to electrostatic repulsion between the positively charged clay–chitosan complex particles. This result is in good agreement with that for the kaolinite modified with the synthetic cationic PDADMAC [34]. Hence, interaction of anionic PHum with kaolinite enhances the slippage of the negatively charged clay particles and reduces the rheological characteristics of the resulting material by 0.5–1.0 orders of magnitude compared to the original clay. Contrastingly, cationic chitosan binds the kaolinite particles into the physical network via electrostatic interaction, thereby increasing the mechanical parameters of kaolinite by 0.5–1.5 orders of magnitude. The rheological characteristics for the original and polymer-modified kaolinite are listed in Table 2. The parameters are represented by a mean value obtained from the triplicate experiment for each system. The next step was the development of natural ameliorants that combine clay-strengthening and biological activity. For this, non-stoichiometric IPCs were formed via cooperative electrostatic interactions between PHum and chitosan as described above. The compositions of the IPC modifiers are shown in Table 1. The IPC content in the mixed kaolinite–IPC system was ω = 0.25 wt.% every time. At this fixed IPC content, the transition from IPC 1 to IPC 4 was accompanied by the growth in the chitosan fraction in the polycomplex.

The rheological characteristics of IPC-modified kaolinite are presented in Table 2. Modification of kaolinite with both cationic and anionic IPCs was accompanied by the growth in the rheological characteristics of clay. Note that the increase in rheological parameters was in parallel with moving from anionic IPC 1 (–) to cationic IPC 4 (+), i.e., with raising the chitosan content in the polycomplexes. Hence, the reinforcing effect of cationic chitosan in the IPC prevails over the loosening effect of anionic PHum.

**Figure 5:**
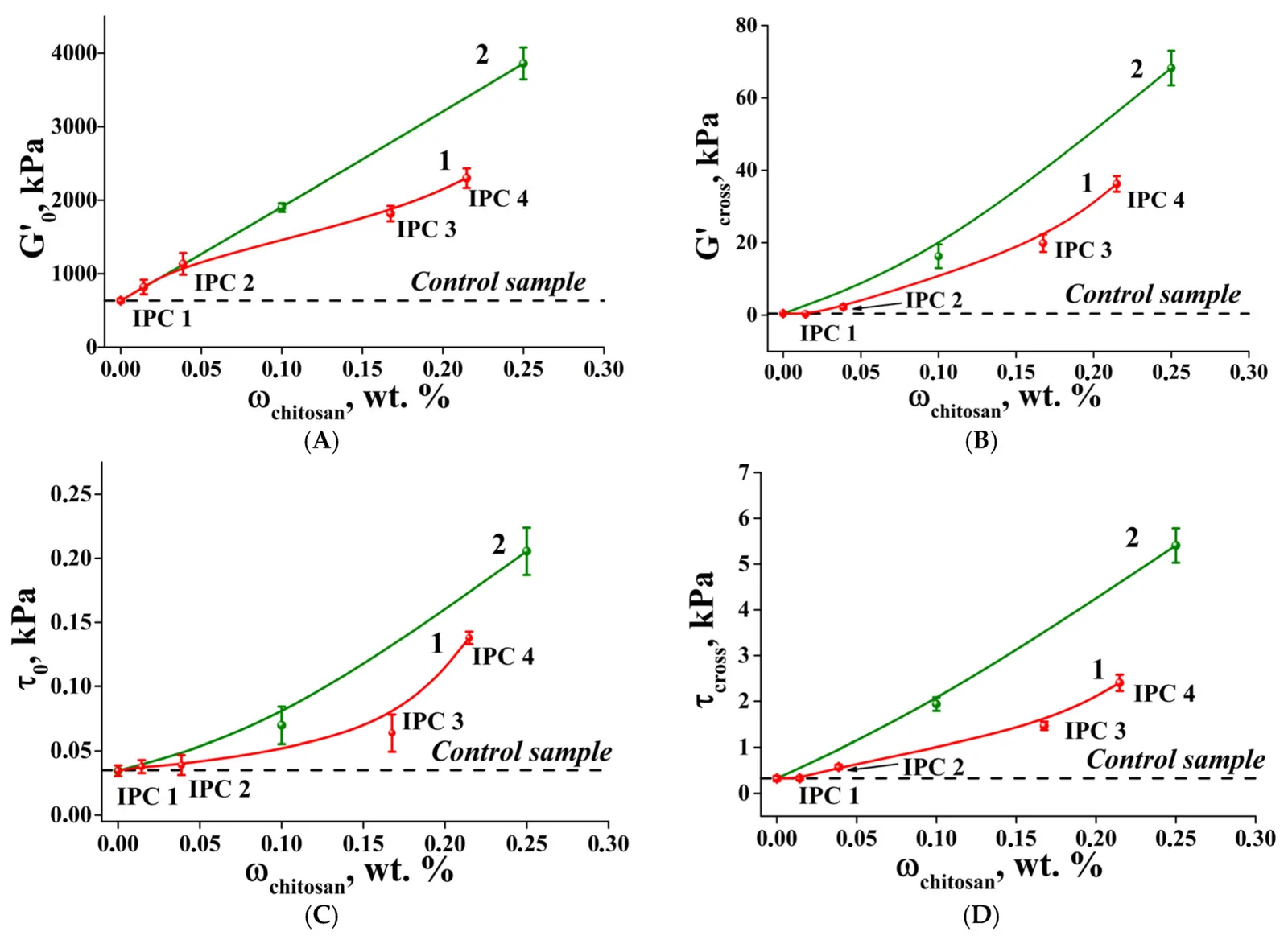
Dependences of G′_0_ (**A**), G′_cross_ (**B**), τ_0_ (**C**) and τ_cross_ (**D**) on chitosan content in kaolinite, ω_chitosan_. Curves 1 correspond to kaolinite samples modified with the interpolyelectrolyte complexes (IPCs) (systems “kaolinite—IPC-solution”), curves 2 to kaolinite samples modified with the individual chitosan (systems “kaolinite—chitosan-solution”). Horizontal dashed lines indicate the corresponding value of the reference kaolinite–acetate-phosphate buffer-saturated sample (control sample). Hereinafter error bars indicate the confidence intervals at *p* < 0.05.

In order to highlight the strengthening effect of chitosan, in Figure 5 (curves 1) the dependences of the rheological parameters on the content of chitosan introduced into kaolinite as part of the four IPCs described above are shown, while control curves 2 show the dependences for kaolinite–chitosan samples, reproduced from Figure 4. In Figure 5, the x-axis reflects the content of chitosan in the resulting kaolinite–polymer mixture.

As follows from the data in Figure 5, all rheological parameters, G′_0_, G′_cross_, τ_0_ and τ_cross_, are lower for the kaolinite–IPC system (curves 1) compared to the control kaolinite–chitosan system (curves 2). Probably, this decrease is due to the rheological reduction effect caused by anionic PHum. Formally, the overall decrease in rheological parameters for the IPC systems is quantified as the summation of the positive effect of chitosan and the negative effect of PHum (see curves 1 and 2 in Figure 4). Thus, the rheological characteristics of IPC-modified clay are mainly controlled by the cationic component chitosan, while PHum, as an anionic component, has no significant effect on them.

To explain this behavior of chitosan–PHum IPCs, the ability of polycomplexes to enter into exchange reactions was taken into account [52,53]. In kaolinite–IPC samples, anionic clay particles compete with anionic PHum for electrostatic binding of cationic chitosan, resulting in a redistribution of chitosan macromolecules between the two anionic components. The final step is likely the formation of a kaolinite–chitosan–PHum ternary complex. In this complex, chitosan ensures the binding of clay particles into a physical network, which improves the clay’s mechanical properties. Anionic biologically active PHum, involved in the ternary complex, has no noticeable effect on the rheological behavior of the material.

## 4. Conclusions

The complexation of natural PEs (cationic chitosan and anionic PHum) was studied. IPCs resistant to phase separation were shown to be formed at Q ≤ 0.3 and Q ≥ 3 where Q = [${\mathrm{NH}}_{3}^{+}$]/[COO^−^] is the ratio between the positively charged amino groups of chitosan and negatively charged carboxylic groups of PHum.

Modification of kaolinite with a minor (not more than 0.5 wt.%) amount of natural cationic chitosan resulted in an increase in the initial storage modulus G′_0_ corresponding to the linear viscoelastic region and the storage modulus G′_cross_ corresponding to the “flow point” by 0.5–1.5 orders of magnitude. Treatment of kaolinite with the same amounts of PHum was accompanied by a decrease in these characteristics by 0.5–1.0 orders of magnitude.

The reinforcing effect of cationic chitosan on anionic kaolinite was due to electrostatic interaction between both components resulting in the formation of a cooperative physical network. In contrast to this, interaction of anionic Hums with kaolinite enhances the sliding of the clay platelets and decreases the rheological characteristics of the material.

PHum is highly requested in agriculture due to its biological activity. Cationic polymers, including chitosan, have been proposed to improve the quality of degraded soils [24,54,55]. It seems attractive to combine biostimulating and stabilizing properties in one ameliorant. For this purpose, chitosan/PHum IPCs, both anionic with Q ≤ 0.3 and cationic with Q ≥ 3, were tested as modifying agents for kaolinite.

Modification of kaolinite with 0.25 wt.% IPCs was accompanied by an increase in the mechanical characteristics of the clay. This result evidenced that the reinforcing action of cationic chitosan in IPCs dominated over the weakening action of the anionic PHum. In other words, the treatment of kaolinite with IPCs preserved the reinforcing effect of chitosan and suppressed the loosening of the clay by PHum, which, however, can act as a biostimulating agent. This allows us to combine stabilizing and biostimulating properties in one chitosan–PHum IPC ameliorant.

The behavior of chitosan/PHum IPCs in relation to kaolinite was explained in terms of exchange reactions, resulting in a redistribution of cationic chitosan macromolecules between the anionic PHum and kaolinite. Ultimately, a kaolinite–chitosan–PHum ternary complex is formed, which improves the mechanical properties of the clay, while the anionic PHum has little effect on the clay’s rheological behavior.

These results are consistent with earlier results concerning the modification of kaolinite by (synthetic PDADMAC)/(natural PHum) IPCs [34] and substantiate the possibility of using the completely natural and biocompatible chitosan/PHum IPCs as environmentally friendly and promising combined ameliorants.

## Figures and Tables

**Table 1:** Composition and electrophoretic mobility (EPM) of non-stoichiometric chitosan/PHum IPCs.

IPCs		Q = [${\mathrm{NH}}_{3}^{+}$]/[COO^−^]	Weight Ratio Chitosan/PHum, %	EPM, (μm/s)/(V/cm)
Anionic	IPC 1 (−)	0.1	5.8/94.2	−2.1 ± 0.3
	IPC 2 (−)	0.3	15.5/84.5	−2.0 ± 0.2
Cationic	IPC 3 (+)	3.3	67/33	1.7 ± 0.2
	IPC 4 (+)	10.0	85.9/14.1	2.3 ± 0.2

**Table 2:** Rheological characteristics of the kaolinite and kaolinite–polymer samples. Polymer content—0.25 wt.%, pH 5.3 ± 0.1.

No.	Sample	Parameter, kPa			
		G′_0_	G′_cross_	τ_0_	τ_cross_
1	Kaolinite	634 ± 28	0.44 ± 0.02	0.035 ± 0.004	0.33 ± 0.05
2	Kaolinite–chitosan	3860 ± 256	68.3 ± 4.8	0.200 ± 0.038	5.40 ± 0.68
3	Kaolinite–Phum	17.7 ± 6.1	0.10 ± 0.01	0.001 ± 5·10^−4^	0.09 ± 0.01
4	Kaolinite–IPC 1 (−)	819 ± 99	0.30 ± 0.05	0.037 ± 0.005	0.33 ± 0.04
5	Kaolinite–IPC 2 (−)	1134 ± 148	2.3 ± 0.3	0.039 ± 0.008	0.58 ± 0.05
6	Kaolinite–IPC 3 (+)	1817 ± 105	19.9 ± 2.4	0.064 ± 0.014	1.46 ± 0.09
7	Kaolinite–IPC 4 (+)	2300 ± 133	36.3 ± 2.2	0.138 ± 0.005	2.40 ± 0.18

## Data Availability

Dataset available on request from the authors.

## Abbreviations

The following abbreviations are used in this manuscript:

PHum	Potassium humate
PE	Polyelectrolyte
IPC	Interpolyelectrolyte complex
PDADMAC	Polydiallyldimethylammonium chloride
EPM	Electrophoretic mobility
